# Is the Use of Personality Based Psychometrics by Cambridge Analytical Psychological Science's “Nuclear Bomb” Moment?

**DOI:** 10.3389/fpsyg.2021.581448

**Published:** 2021-01-29

**Authors:** Eric C. Prichard

**Affiliations:** School of Social and Behavioral Sciences, University of Arkansas at Monticello, Monticello, AR, United States

**Keywords:** Cambridge analytica, personality psychology, psychometrics, big data, ethics

As J. Robert Oppenheimer witnessed the first nuclear explosion in history, he famously quoted the Bhagavad Gita: “Now I am become death, the destroyer of worlds” (Temperton, 2017). Scientists responsible for the creation of the atomic bomb would now have to live with the consequences of bringing about a weapon that could destroy humanity. Whether the reason for participating in the creation of these weapons was a desire to stop Hitler, patriotism, or a love of the scientific problem of splitting the atom, the consequence of the creation of the atomic bomb has been a world that is forever change and more dangerous than it had been prior to the Second World War.

In this article, I argue that psychological science is having its own nuclear moment. In the first part, I discuss the role Psychological Science played in the Cambridge Analytica scandal. I argue that the unleashing of psychometrics which can be used by the Military Industrial Complex and big data companies has contributed to a world that is more dangerous and forever changed. In the second part, I make recommendations for what psychological scientists can do about it.

## I Am Become Death, Destroyer of Democracies

In 2018, Carole Cadwalladr wrote an extensive article for the U.K.'s *Guardian*, in which she interviewed former Cambridge Analytica employee turned whistleblower Christopher Wylie (Cadwalladr, 2018). The article details how Cambridge Analytica and its parent company SCL (formerly Strategic Communications Laboratory) worked for the Brexit Campaign, Trump Campaign, various global political misinformation campaigns, and had contracts with the U.S. State Department. A big emphasis of the article is how Cambridge Analytica/SCL unethically pulled data from Facebook and created psychological profiles of 230 million Americans. What should alarm psychological scientists, however, are the central roles that academic psychology and personality psychology research played in the creation of what Wylie referred to as “Steve Bannon's psychological warfare mindfuck tool” (Cadwalladr, 2018).

In relation to the press work by Cadwalladr (2018), several academic sources from outside of psychology have noted the central role of psychological scientists in the scandal. Richterich (2018) details a history of Cambridge Analytica that closely coincides with that reported by Cadawalldr. According to both sources, Cambridge University's Psychometrics Center played a pivotal role. Cambridge researcher David Stillwell developed an app for Facebook called “My Personality” in 2007 and was joined on the project by then Cambridge Ph.D. student Michal Kosinski in 2009. Kosinski and Stillwell were able to get millions of people to take a questionnaire on Facebook based on the “big five” traits. Another Cambridge employee, Aleksandr Kogan, attempted to mediate a deal between the developers of “My Personality” and SCL. At this point, according to Cadwalladr (2018), Kosinski asked for $500,000 for the data. Kogan decided to replicate the results himself and set up a company called Global Science Research (GSR). In 2014 Kogan launched “thisisyourdigitallife,” which was an ostensible personality app on Facebook. However, the app also allowed GSR to pull data from 160 other people for every one person that took the test. Once the data were pulled, personality profiles for millions of people were constructed and Cambridge Analytica was ready to turn psychometrics into psychological weapons.

Berghel (2018) and Heawood (2018) both argue that the massive amounts of personality data allowed Cambridge Analytica to engage in mass manipulation campaigns via enhanced microtargeting. The authors describe microtargeting as targeted messaging for specific groups. Heawood (2018) acknowledges that microtargeting has origins in advertising and is not a bad thing *per se*. It has long been used to advertise specific goods and services to specific audiences. For example, if one is trying to sell a specific beverage, it might be advertised by an athlete on an ad in the sports section of a newspaper and an actress on an ad in the entertainment section. The specific type of microtargeting criticized by Berghel and Heawood which has been used on social media platforms by Cambridge Analytica to sway elections. In this case, the micotargeting was based on personality characteristics and other useful data obtained via Facebook. Worryingly, it was obtained by a private company that was willing to manipulate people, based on their psychological profiles, for the highest bidder. Berghel (2018) notes that this threatens democracy by increasing the spread and efficiency of international propaganda. Heawood (2018) argues that social media based microtargeting seemingly personalizes propagandistic messages. He argues that this kind of messaging would make people skeptical if it to appear were on a billboard, but is ismore readily accepted when it appears on social media and is shared by known contacts. Social media messaging also protects micortargeted propaganda messages from being subject subject to normal criticism in the marketplace of ideas. This creates what Heawood (2018) called “pseudo-public” messaging. This means claims that were once had to be stated publicly, such as political claims on a billboard, are now share privately through electronic media, protecting those claims from public scrutiny.

More chillingly, Cambridge Analytica made a pitch in 2014 to a Russian oil firm called Lukoil about the application of psychometric data to microtargeting in elections (Cadwalladr, 2018). The data pitched regarded the behavior of American voters. What interest would an oil company have in the behavior of voters in a foreign country? Why would they be interested in election disruption? According to Cadwalladr, Wylie described Cambridge Analytica as an unaccountable MI6 that was completely mercenary and willing to sell out to anyone who pays, even when selling out meant disrupting the democratic process across the world by actively spreading targeted misinformation that reinforces the propaganda of authoritarian politicians.

It's easy to point to SCL/Cambridge Analytica as bad actors. One could argue that scientists merely do research and knowledge can be used for good or bad, but the responsibility lies with the user. That's a comforting argument coming from an office in an ivory tower. It's quite a different thing to argue science is a value neutral search for truth when one amidst smoldering ruins of cities destroyed by advanced weapons technology or living in a crumbling democracy being torn apart by propaganda fueled factionalism. Psychological science is at the heart of the technology Cambridge Analytica used. And psychologists, as a community, need to be part of society's response to the widespread use of targeted propaganda spreading through social media.

## What Psychological Scientists Can Do

Elsewhere, I have argued that psychologists need to make careful consideration of the moral implications of their research when it has an effect on policy (Prichard, 2019). I reproduce the “Matrix of Moral Considerations” from that publication as Table 1.

**Table 1:** Matrix of Moral Considerations (Prichard, 2019).

	**Individuals as objects of an action with moral value**	**Communities as objects of an action with moral value**
Moral obligations as an individual	Do the presentation of theory and the subsequent policy recommendation treat the individuals who will be affected as ends in and of themselves?	Do the presentation of theory and the subsequent policy recommendation treat the communities which will be affected as ends in and of themselves?
Moral obligations as a member of a community	How do the presentation of theory and the subsequent policy recommendation contribute toward the effects of the collective action of the community/communities I am representing on individuals?	How do the presentation of theory and the subsequent policy recommendation contribute toward the effects of the collective action of the community/communities I am representing on communities?

I argue that when psychologists apply their research to policy, there are four considerations they should make.

For the purpose of this moral analysis, I will argue that the policy in question is the policy of using microtargeting to manipulate voters and subvert democratic processes. This may be the policy of a political campaign, a private company, or a state actor. The application of theory in question is the application of psychometrics and personality theory. I intend to show that the policy fails at every level of the matrix and I will make a recommendation regarding what psychologists can do.

First and foremost, the data rights of individuals who had their data pulled were violated. As Berghel (2018) notes, many of the people who took the “thisisyourdigitallife” quiz were unaware that the personal data of their friends and family members would be taken by GSR. Virtually all of those friends and family members would have been unaware. And everyone whose data was used to influence campaigns such as the 2016 U.S. Presidential Election would have been unaware that their personality data were being used in a mass influence campaign. If psychological tools are going to be used in this manner, psychologists have an obligation to fight for data rights. By data rights, I do not mean the protection of patient information such as is required by the Health Insurance Portability and Accountability Act (HIPAA) or the data security measures required by psychological scientists doing academic research. By data rights, I mean the right of an individual to access their data collected by private corporations such as Facebook and the right to request and receive information regarding who is using their data and how their data are being used. APA and APS, among other organizations, should be front and center in the fight for data rights as individual rights. People are horrified by the idea of medical experiments being performed on patients without their consent. If major scientific organizations would be willing to draw stronger parallels between the use of data obtained by companies like Facebook with data collected for medical or academic purposes, then it might give some direction to policy makers regarding the ways in which data can be protected.

Second, a democracy is a community. The use of microtargeted information to subvert democracy is an attack on that community. Psychological scientists should condemn these attacks on democracy. But that is not enough. As teachers, professors of psychology must make anti-propaganda skills part of the curriculum. They must teach students how to identify and defend themselves against persuasion techniques. Cyberwar and psychological war are here to stay. The knowledge required to resist propaganda is essential in this day and age, and psychologists can play a big role in fostering this through education. This may even mean adding units, as part of the standard curriculum, to introductory psychology courses about the ways social media is used to misinform and manipulate the public. This material would likely be introduced alongside traditional lessons about persuasion taught during units covering social psychology.

As an individual psychologist, I have a choice about who I will work for and to whom I will give my data. I encourage psychologists to think twice about taking money from the military or industry without assurances that the rights of individuals and communities will be protected. Career advancement and financial opportunities can be tempting. So can be the intellectual challenges private and government projects provide. However, Wylie's story is a cautionary tale (Cadwalladr, 2018). Wylie lost sight of the big picture because of the exciting intellectual challenges of his work. But he now shares culpability for the ways in which his research was used.

Finally, psychological scientists need to have a concern for the collective moral action of our community. We have powerful lobbying arms in the form of associations such as APA. We hold positions in academia and the government. We are recognized experts. We have journals and access to media. There is no reason that psychological science collectively cannot pool its resources and fight for data rights. Psychology can make a mass push to teach defense against propaganda. Psychological bodies can be firm on their stances and in their negotiations with government and business. We should not allow our science to be used to violate human rights and we should be vocal and protest when such attempts are made. If the community collectively fails or allows itself to be divided because of the alluring spoils of military and private contracts, then we all share some responsibility.

The atom bomb changed the world forever. So has the use of psychometrics to mass manipulate people through social media. Physics had to grapple with its role in making the bomb. We have to grapple with our role in making Cambridge Analytica. How psychology as a community chooses to do so may play a big role in whether we become party to the destroyers of the democratic world or responsible agents in the defense of democracy.

## Author Contributions

The author confirms being the sole contributor of this work and has approved it for publication.

## Conflict of Interest

The author declares that the research was conducted in the absence of any commercial or financial relationships that could be construed as a potential conflict of interest.
